# Synaptic potentiation in the nociceptive amygdala following fear learning in mice

**DOI:** 10.1186/1756-6606-6-11

**Published:** 2013-03-01

**Authors:** Ayako M Watabe, Toshitaka Ochiai, Masashi Nagase, Yukari Takahashi, Masaru Sato, Fusao Kato

**Affiliations:** 1 Department of Neuroscience, Jikei University School of Medicine, Tokyo 105-8461, Japan; 2 Precursory Research for Embryonic Science and Technology (PRESTO), Japan Science and Technology Agency, Kawaguchi, Saitama 332-0012, Japan; 3 Department of Pathophysiology and Therapeutics, School of Pharmacy and Pharmaceutical Sciences, Hoshi University, Tokyo 142-8501, Japan; 4 Department of Anesthetics, Jikei University School of Medicine, Tokyo 105-8461, Japan; 5 Nagoya University Graduate School of Medicine, Nagoya 466-8550, Japan

**Keywords:** Central amygdala, Fear conditioning, Synapse, Plasticity, Potentiation, Mouse, Nociception

## Abstract

**Background:**

Pavlovian fear conditioning is a classical form of associative learning, which depends on associative synaptic plasticity in the amygdala. Recent findings suggest that the central amygdala (CeA) plays an active role in the acquisition of fear learning. However, little is known about the synaptic properties of the CeA in fear learning. The capsular part of the central amygdala (CeC) receives direct nociceptive information from the external part of the lateral parabrachial nucleus (lPB), as well as highly processed polymodal signals from the basolateral nucleus of the amygdala (BLA). Therefore, we focused on CeC as a convergence point for polymodal BLA signals and nociceptive lPB signals, and explored the synaptic regulation of these pathways in fear conditioning.

**Results:**

In this study, we show that fear conditioning results in synaptic potentiation in both lPB-CeC and BLA-CeC synapses. This potentiation is dependent on associative fear learning, rather than on nociceptive or sensory experience, or fear memory retrieval. The synaptic weight of the lPB-CeC and BLA-CeC pathways is correlated in fear-conditioned mice, suggesting that fear learning may induce activity-dependent heterosynaptic interactions between lPB-CeC and BLA-CeC pathways. This synaptic potentiation is associated with both postsynaptic and presynaptic changes in the lPB-CeC and BLA-CeC synapses.

**Conclusions:**

These results indicate that the CeC may provide an important locus of Pavlovian association, integrating direct nociceptive signals with polymodal sensory signals. In addition to the well-established plasticity of the lateral amygdala, the multi-step nature of this association system contributes to the highly orchestrated tuning of fear learning.

## Background

The amygdala plays a key role in fear learning by attaching emotional value to various types of sensory input [1,2]. In Pavlovian fear conditioning, a previously emotionally neutral conditioned stimulus (CS) acquires the ability to elicit defensive responses when it is presented in conjunction with an aversive unconditioned stimulus (US). While this associative learning has been considered to occur within the lateral nucleus of the amygdala (LA), growing evidence suggests that the central nucleus of the amygdala (CeA), which was previously considered to function as a passive relay to downstream targets that mediate fear responses, also plays an active role in the acquisition of fear learning [3,4]. These findings suggest that both the LA and CeA play crucial roles in associating the US with the CS, and that neuronal plasticity in the LA and CeA may regulate associative fear learning in a cooperative manner.

CeA neurons are predominantly GABAergic inhibitory neurons, and can be divided into at least three distinct subnuclei; lateral, capsular and medial (CeL, CeC and CeM) [5-8]. Recent reports suggest that the CeL and/or the CeC tonically inhibit CeM, and fear learning induces disinhibition of this microcircuit [9-11]. However, while these studies have clearly demonstrated changes in CeL/CeC activity during and after the acquisition of fear learning using unit recording *in vivo*, little is known about how these changes are regulated synaptically.

The CeC has been termed the *nociceptive amygdala*. This area receives major input directly from the external part of the pontine lateral parabrachial nucleus (lPB), which is a major target of nociceptive superficial layers of the dorsal horn [12-16]. The CeC also receives excitatory input from the basolateral nucleus of the amygdala (BLA), which transmits polymodal sensory information, including nociception, from thalamic and cortical regions [17-21]. Together, these findings suggest that the CeC may also be an important locus of CS-US association by integrating BLA and lPB signals.

Despite recent demonstration of nociception-induced plasticity at BLA-CeC and lPB-CeC synapses [14,22-25] and of an N-methyl-D-aspartate (NMDA) receptor-independent presynaptic form of long-term potentiation (LTP) at lPB-CeL synapses [26], it is still unclear how the CeC is involved in fear learning, which uses nociceptive inputs as US.

Here we found that the synaptic weights of both lPB-CeC and BLA-CeC pathways were increased after fear learning in an associative manner, and were mediated by both presynaptic and postsynaptic mechanisms. These results suggest that the CeC may constitute another locus of CS-US association, in addition to the LA, in fear learning.

## Results

### Synaptic potentiation at lPB-CeC and BLA-CeC synapses after fear learning

**Figure 1:**
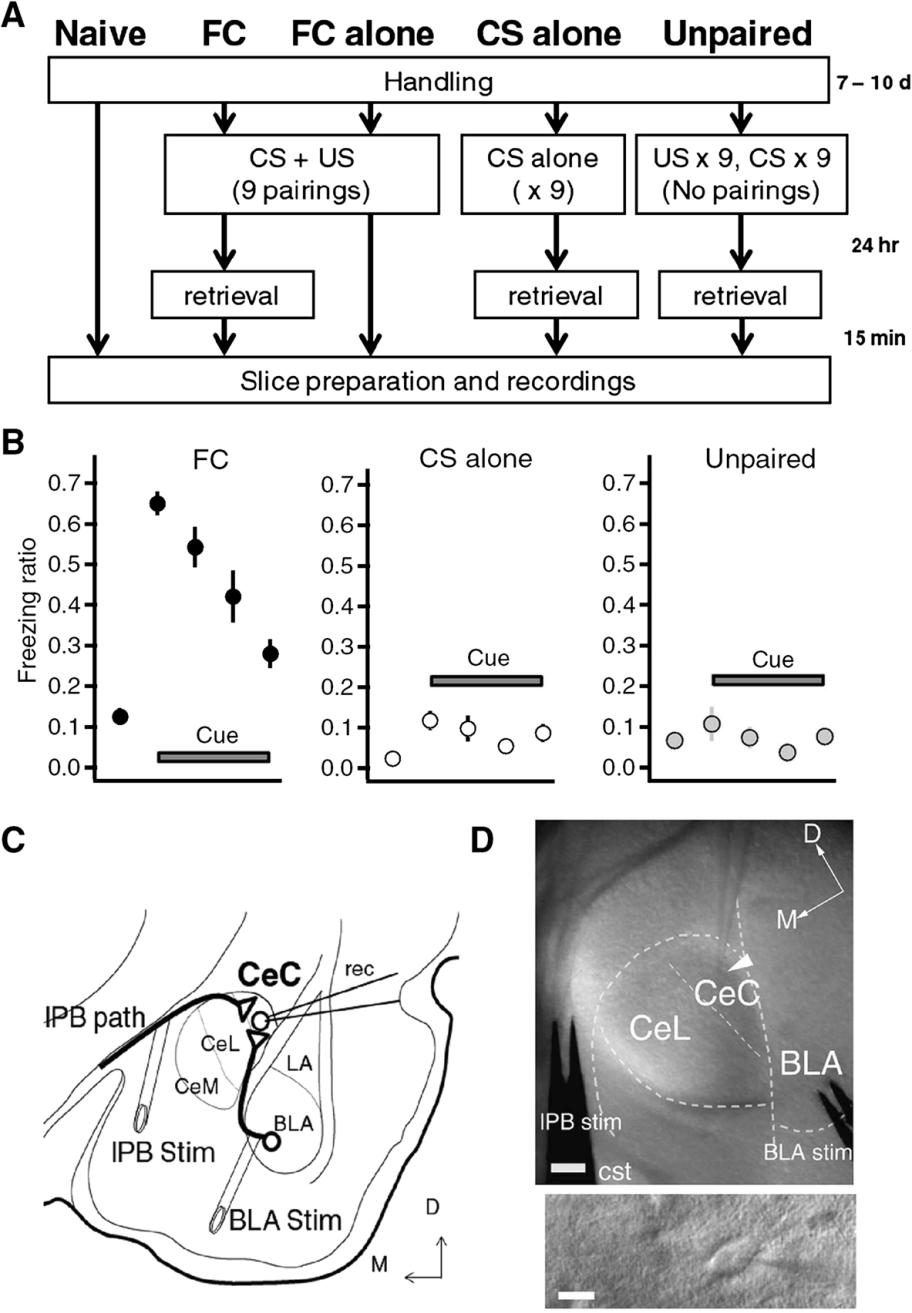
**Experimental setup to examine lPB-CeC and BLA-CeC EPSCs following fear learning. A**, Experimental schedules for the five different mice groups. FC, fear conditioning; CS, conditioned stimulus; US, unconditioned stimulus. **B**, Freezing time ratio during retrieval. The first points represent the freezing ratio during the 2-min baseline period in the chamber, while the 2^nd^ to 5^th^ points corresponds to 1–30 s, 31–60 s, 61–90 s and 91–120 s after the onset of the CS presentation. **C**, Recording configuration for lPB-CeC and BLA-CeC EPSCs. **D**, Oblique illumination optical images showing electrode placement (tip of a recording electrode indicated with an arrowhead) and CeC cells (bottom). Scale bars are 100 μm (top) and 10 μm (bottom).

**Figure 2:**
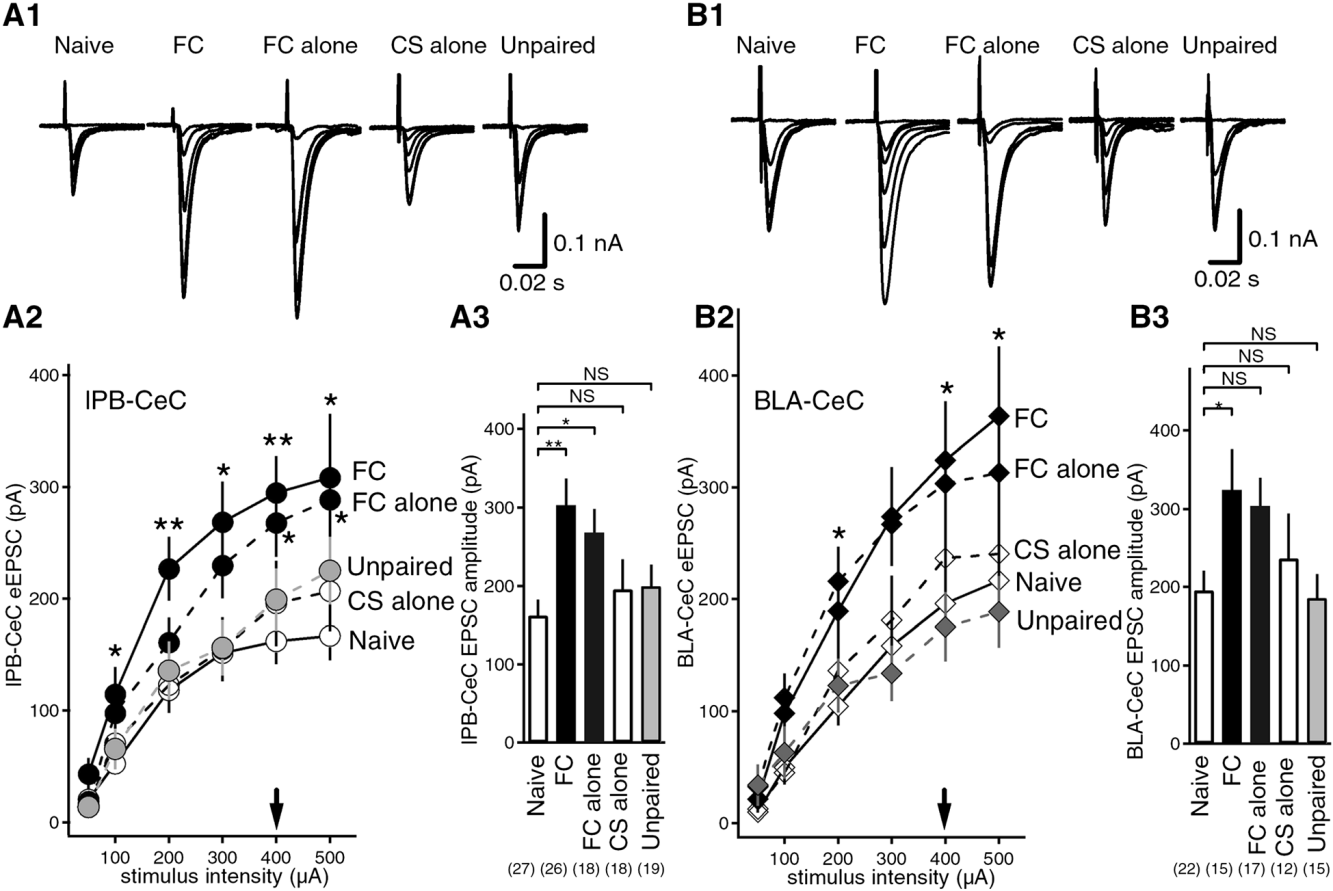
**lPB-CeC and BLA-CeC EPSCs in the five experimental groups. A1**, Averaged traces of eight consecutive lPB-CeC EPSCs with increasing stimulus intensities. **A2**, Relationships between stimulus intensity and lPB-CeC EPSC amplitude, expressed as mean ± SEM. lPB-CeC synapse in FC mice (filled circle, solid line; n = 13–26) and FC alone mice (filled circle, dashed line; n = 18) revealed significantly enhanced synaptic transmission compared with naive mice (open circle, solid line; n = 18–27). In comparison, CS alone mice (open circle, dashed line; n = 16–18) and unpaired mice (grey circle, dashed line; n = 18–20) showed indistinguishable lPB-CeC EPSC amplitudes. *p < 0.05, **p < 0.01, analyzed with *post hoc* Dunnett’s *t*-test following ANOVA. **A3**, CeC EPSC amplitudes evoked by lPB stimuli of 400 μA intensity. **B1***,* Averaged traces of eight consecutive BLA-CeC EPSCs with increasing stimulus intensities. **B2,** Relationships between stimulus intensity and BLA-CeC EPSC amplitude, expressed as mean ± SEM. BLA-CeC synapse in FC mice (filled diamond, solid line; n = 10–17) and FC alone mice (filled diamond, dashed line; n = 17) revealed significantly enhanced synaptic transmission compared with naive mice (open diamond, solid line; n = 18–27). On the other hand, CS alone mice (open diamond, dashed line; n = 11–14) and unpaired mice (grey diamond, dashed line; n = 15–16) showed indistinguishable BLA-CeC EPSC amplitudes. *p < 0.05, analyzed with *post hoc* Dunnett’s *t*-test following ANOVA. **B3**, CeC EPSC amplitudes evoked by BLA stimuli of 400 μA intensity (bottom right).

Previous studies have reported that lPB-CeC synapses and BLA-CeC synapses are morphologically distinct. Most lPB-CeC synapses are asymmetric shaft synapses formed on proximal dendrites, and are typically large in size. In contrast, most BLA-CeC synapses are spinal synapses on more distal dendrites, and tend to be smaller in size [27]. Thus, it is possible that information from the lPB and BLA pathways can be integrated by CeC neurons in a cooperative manner during the fear learning process. To examine this possibility, we first compared the input–output relationship in both lPB-CeC and BLA-CeC synapses in brain slices obtained from the following five groups of mice: naive group, fear-conditioned (FC) group, FC alone group, CS alone group, and unpaired group (Figure 1A). We examined the behavior of these mice (Figure 1B) and then analyzed the effects of the different procedures on evoked EPSCs recorded at a holding potential of −60 mV in the presence of 100 μM picrotoxin at both lPB-CeC synapses and BLA-CeC synapses *in vitro* (Figure 1C, D and 2). Fear conditioning consisted of nine pairings of tones as CS and foot shocks as US in a conditioning chamber. The mice then completed a retrieval test 24 h later in a retrieval chamber which has distinct feature from a conditioning chamber, and were subjected for slice preparation approximately 15 min after the end of the retrieval test (Figure 1A). Mice in the FC alone group were subjected to fear conditioning but were not given the retrieval test session the next day. Mice in the CS alone group were not given conditioning, and only received the CS, and were then subjected to testing 24 h later. Mice in the unpaired group were given US immediately after getting into the chamber, but the CS was given much later, so that no CS-US association formed (Figure 1A). During the retrieval test, the mice in the FC group showed robust freezing behavior (65.0 ± 2.1%) during the first 30-s period of CS exposure (pre-CS; 12.5 ± 2.0%, n = 16, *p* < 0.001), while the mice in the CS alone and unpaired groups showed a much lower level of freezing behavior during the first 30-s period of CS exposure (CS alone group: 11.66 ± 2.35% and 2.25 ± 0.53%, n = 5, *p* = 0.015; unpaired group: 10.66 ± 4.20 and 6.58 ±1.71%, n = 5, *p* = 0.408; for the first 30 s of CS and pre-CS, respectively; Figure 1B). We then prepared coronal brain slices containing the amygdala (Figure 1C, D) approximately 15 min after the end of retrieval for the FC, CS alone and unpaired group, 24 h after conditioning for the FC alone group, and after handling-habituated only for the naive group (Figure 1A). Significant intergroup differences in evoked excitatory postsynaptic currents (EPSCs) at the lPB-CeC and BLA-CeC synapses were found (Figure 2).

Dunnett’s *post hoc* test following one-way ANOVA to compare multiple groups with a control naive group revealed that the EPSCs at the lPB-CeC synapse had a significantly larger amplitude in FC and FC alone mice, but not in the CS alone or unpaired mice compared with naive mice (100 μA: [ANOVA] F_4, 102_ = 1.933, *p* = 0.111, naive vs. FC, *p* = 0.034; 200 μA: F_4, 102_ = 4.247, *p* = 0.003, naive vs. FC, *p* = 0.001; 300 μA: F_4, 83_ = 3.466, *p* = 0.011, naive vs. FC, *p* = 0.012; 400 μA: F_4, 98_ = 4.181, *p* = 0.004, naive vs. FC, *p* = 0.002, naive vs. FC alone, *p* = 0.042; 500 μA: F_4, 77_ = 2.942, *p* = 0.026, naive vs. FC, *p* = 0.018, naive vs. FC alone, *p* = 0.042) (Figure 2A2, A3). These results suggest that basal synaptic transmission at lPB-CeC synapses is enhanced after fear learning. In addition, we found that evoked EPSCs at BLA-CeC synapses were also significantly enhanced in a fear learning-dependent manner (Figure 2B). The amplitude of EPSCs at BLA-CeC synapses were also greatly enhanced in FC and FC alone mice, but not in CS alone or unpaired mice compared with naive mice (200 μA: [ANOVA] F_4, 80_ = 2.642, *p* = 0.040, naive vs. FC alone, *p* = 0.020; 300 μA: F_4, 98_=3.222, *p* = 0.017; 400 μA: F_4, 98_ = 4.181, *p* = 0.032, naive vs. FC, *p* = 0.048; 500 μA: F_4, 68_ = 3.009, *p* = 0.024, naive vs. FC, *p* = 0.033) (Figure 2B2, B3). Passive membrane properties, such as resting membrane potential and input resistance, were indistinguishable between the groups (Em: [ANOVA] F_4, 112_=1.441, *p* = 0.225; Ri: F_4, 114_=0.707, *p* = 0.589) (Table 1). These results strongly suggest that basal synaptic transmission at both lPB-CeC and BLA-CeC synapses are enhanced after fear learning. Furthermore, this potentiation is associative, and not primarily dependent on fear retrieval or nociceptive experience *per se*, but rather on fear learning.

**Table 1:** Passive membrane properties of CeC neurons

** Groups**	**Resting membrane potential (mV)**	**Input resistance (MΩ)**
Naive (n = 30)	−62.7 ± 0.7	191.1 ± 17.7
FC (n = 29)	−64.5 ± 1.0	199.6 ± 25.2
FC alone (n = 22)	−62.1 ± 1.8	223.0 ± 33.5
CS alone (n = 18)	−61.2 ± 1.2	169.9 ± 15.9
Unpaired (n = 20)	−61.0 ± 1.3	218.0 ± 26.6

### Presynaptic changes following fear learning at lPB-CeC and BLA-CeC synapses

**Figure 3:**
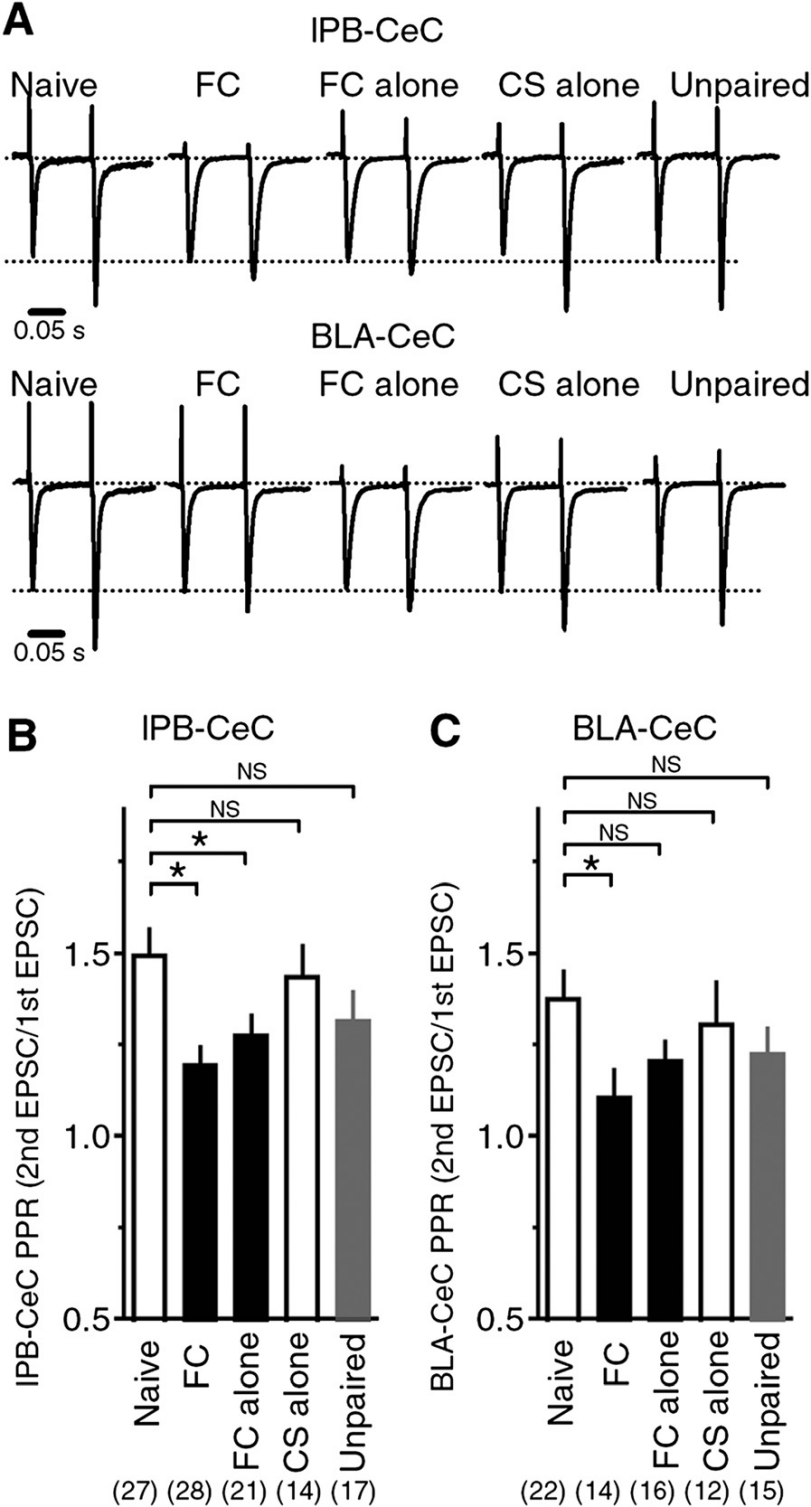
**Paired-pulse ratio in lPB-CeC and BLA-CeC pathways. A**, Representative traces (average of eight consecutive EPSCs) of scaled lPB-CeC EPSCs (top) and BLA-CeC EPSCs (bottom) from each group of mice. **B**, Averaged paired-pulse ratio (PPR) evoked by paired stimuli (100-ms interstimulus interval) in lPB-CeC synapses. Note that the PPR was significantly reduced in FC mice (n = 28) and FC alone mice (n = 21) compared with naive mice (n=27), but CS alone (n = 14) and unpaired (n = 17) mice displayed an indistinguishable PPR compared with naive mice, analyzed with *post hoc* Dunnett’s *t*-test following ANOVA. **C***,* Averaged PPR evoked by paired stimuli (100-ms interstimulus interval) in BLA-CeC synapses. Note that the PPR was significantly reduced in FC mice (n = 28) compared with naive mice (n = 27), but FC alone (n = 21), CS alone (n = 14) and unpaired (n = 17) mice showed indistinguishable PPR compared with naive mice, analyzed with *post hoc* Dunnett’s *t*-test following ANOVA.

To explore the mechanisms underlying the synaptic potentiation observed at the lPB-CeC and BLA-CeC synapses, we investigated the possible involvement of presynaptic mechanisms by measuring the paired-pulse ratio (PPR) of EPSCs, a parameter affected by changes in release probability from the presynaptic terminal [28]. We found that the PPR at lPB-CeC synapses was significantly decreased in the FC and FC alone groups, but not in the CS alone or unpaired groups, compared with naive mice (PPR: 1.50 ± 0.07, 1.20 ± 0.05, 1.28 ± 0.05, 1.44 ± 0.08 and 1.32 ± 0.08; n = 28, 29, 22, 15 and 18 for naive, FC, FC alone, CS alone and unpaired groups, respectively. ANOVA followed by Dunnett’s test: F_4, 102_ = 3.906, *p* = 0.005, naive vs. FC, *p* = 0.002, naive vs. FC alone, *p* = 0.046. Figure 3A, B). The PPR at BLA-CeC synapses was also significantly decreased in the FC group compared with the naive group. The FC alone, CS alone and unpaired groups did not display significant differences (PPR: 1.38 ± 0.07, 1.11 ± 0.07, 1.21 ± 0.05, 1.31 ± 0.11 and 1.23 ± 0.07; n = 22, 14, 16, 12 and 15 for naive, FC, FC alone, CS alone and unpaired groups, respectively. ANOVA followed by Dunnett’s test: F_4, 76_ = 1.992, *p* = 0.104, naive vs. FC, *p* = 0.039; Figure 3A, C). These results suggest that the release probability from presynaptic terminals was increased in both the lPB-CeC and BLA-CeC pathways in fear-conditioned mice, contributing to the synaptic potentiation observed in Figure 2A and B.

### Postsynaptic mechanisms underlying the potentiation of lPB-CeC and BLA-CeC synapses

**Figure 4:**
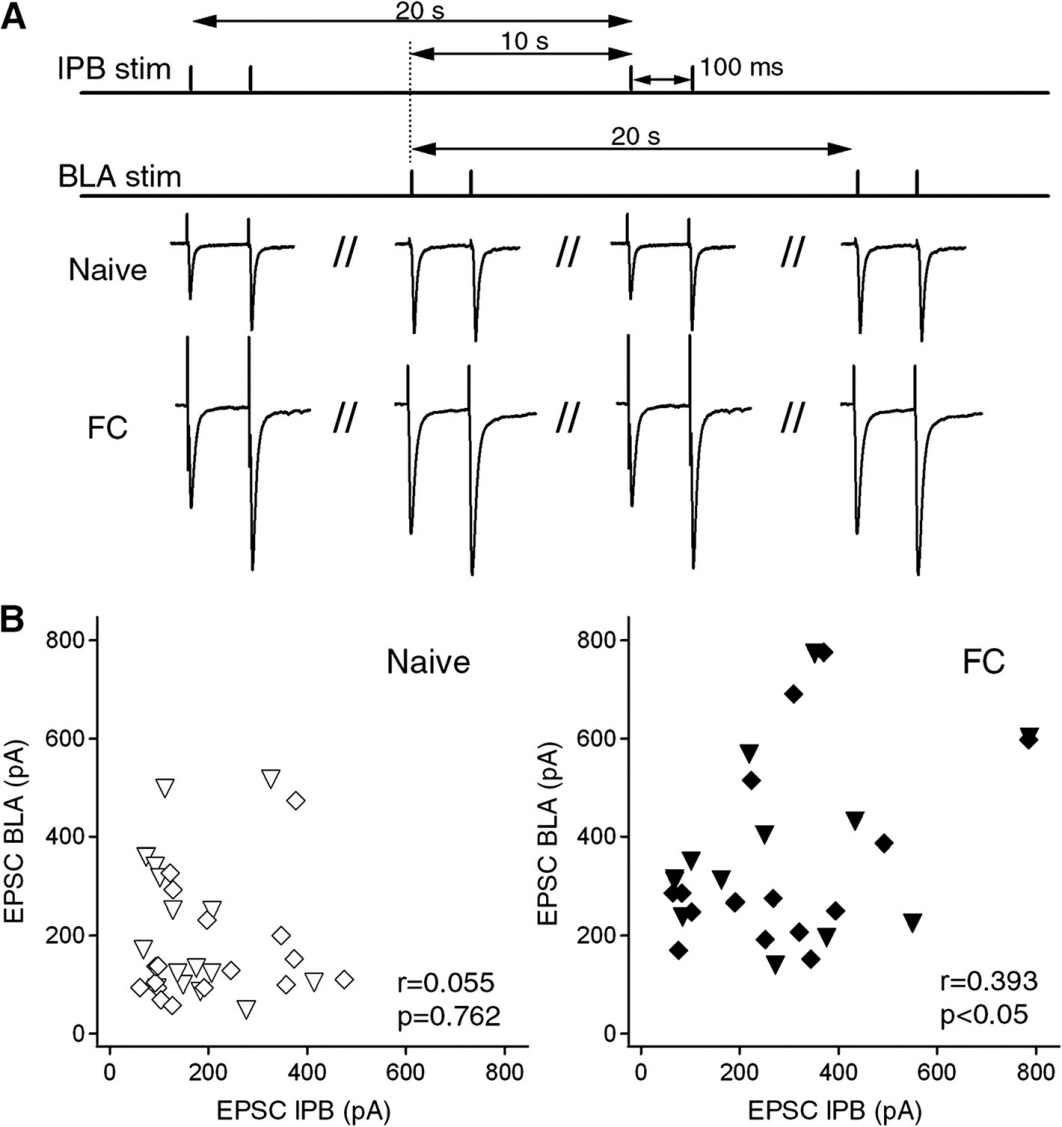
**Correlation between lPB-CeC and BLA-CeC EPSC amplitudes. A**, Experimental design for alternative stimulation of lPB and BLA pathways. **B**, Correlation between EPSC amplitudes of lPB-CeC (abscissa) and BLA-CeC (ordinates) synapses in naive mice (left panel, open symbols; n = 33) and fear-conditioned mice (right panel, filled symbols; n = 29). Each symbol represents data from the same set of neurons of the two pathways; the stimulation intensity of the lPB and BLA pathways was identical for these data. Different symbols represent recordings evoked by 400 μA (diamonds) and 500 μA (inverted triangles) stimulus intensities.

Most, if not all, of the CeC neurons receive excitatory inputs from both the lPB and BLA pathways [25,29]. We therefore examined whether the increase in evoked EPSC amplitude in one of these pathways was associated with a change in the other pathway in individual neurons. To address this, we stimulated the lPB and BLA pathways alternately and compared the amplitude of lPB-CeC EPSCs and BLA-CeC EPSCs recorded with identical stimulus intensities (400 μA–500 μA) (Figure 4A). In naive mice, there was no significant correlation between the lPB-CeC EPSCs and BLA-CeC EPSCs recorded in each CeC neuron (Pearson’s correlation, r = 0.055, *p* = 0.762, n = 33). In contrast, there was a significant correlation between the amplitudes of EPSCs in these pathways after fear learning (r = 0.393, *p* = 0.035, n = 29; Figure 4B). While these results suggest some form of heterosynaptic plasticity might coordinate the changes in the two pathways, another possibility is that the electrical stimuli delivered at the two sites activate partially overlapping sets of axons. To rule out this possibility, we performed “cross-pathway PPR experiments”, in which the lPB-CeC pathway was stimulated 50 ms after the BLA-CeC pathway, and *vice versa,* in naive male C57BL/6 J mice. Cross-pathway PPR is defined as EPSC _2nd BLA of cross-path PPR (lPB-BLA)_/EPSC _1st BLA of single-path PPR (BLA-BLA)_ for lPB effect on BLA pathway, and EPSC _2nd lPB of cross-path PPR (BLA-lPB)_/EPSC _1st lPB of single-path PPR (lPB-lPB)_ for BLA effect on lPB pathway. We found that prior stimulation in one pathway had an almost negligible effect on the EPSC amplitude of the other pathway (for lPB effect on BLA: 0.99 ± 0.08, n=6; BLA effect on lPB: 0.99 ± 0.02, n=6). The conventional PPRs, with 50-ms interval, were 1.82 ± 0.11 and 2.05 ± 0.12 for the BLA (n=6) and lPB (n=6) pathways, respectively, in the same cells obtained before and after the cross-pathway PPR experiments. Taken together, the data indicate that fear learning induces potentiation at both lPB and BLA synaptic inputs onto CeC neurons, and suggest that a type of cooperative postsynaptic interaction occurs between the lPB-CeC and BLA-CeC pathways when their signals are integrated by CeC neurons.

**Figure 5:**
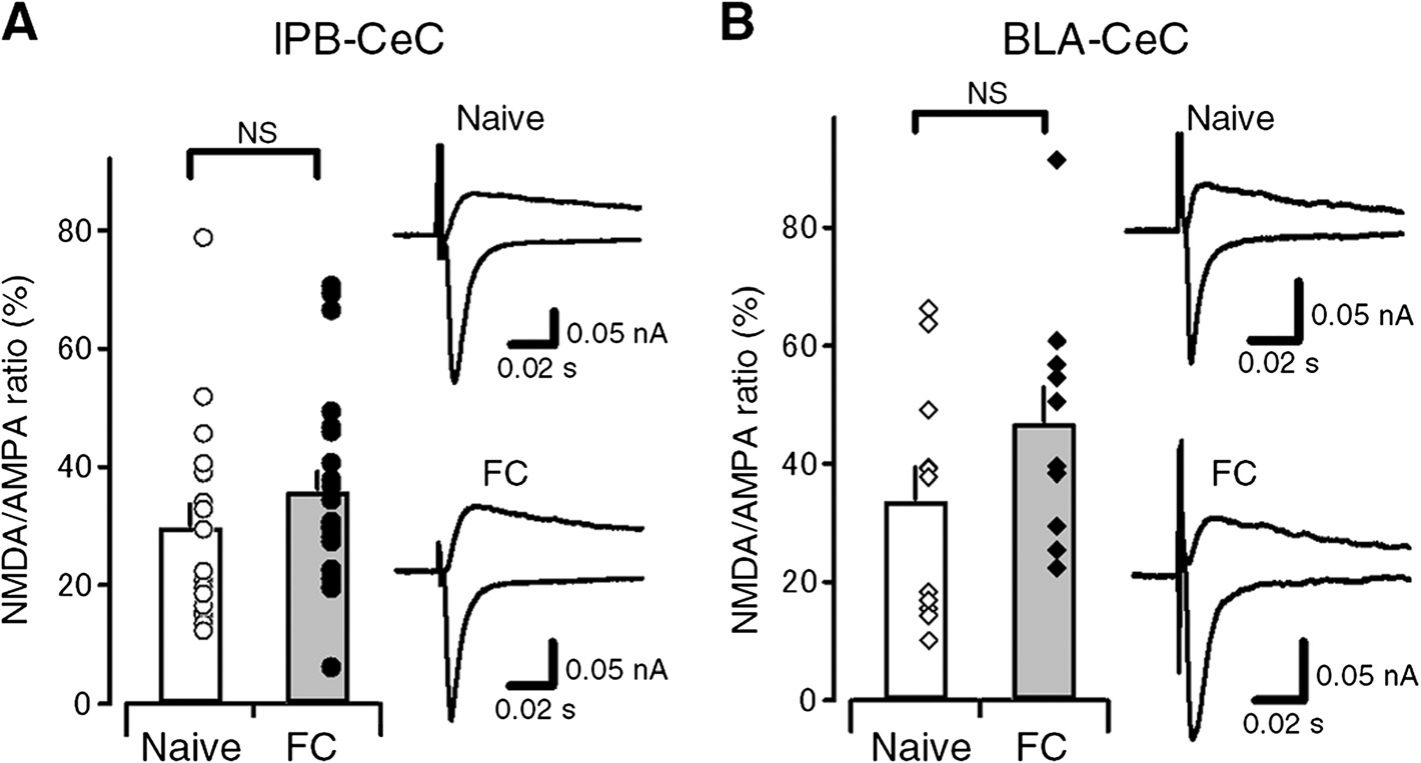
**NMDA/AMPA ratio in lPB-CeC and BLA-CeC synapses. A**, The NMDA/AMPA ratio in naive (open bar; n = 17) and fear-conditioned (filled bar; n = 22) mice at lPB-CeC synapses. Circles indicate the individual ratio for each neuron. Sample traces of NMDA current (upper trace) and AMPA current (lower trace) are superimposed for naive (top) and FC (bottom) mice. The average of 15 consecutive waves (5 min) taken 20–25 min after CNQX application, followed by changing holding potential to Vh = +40 mV, and those taken 5 min before the application of CNQX at a holding potential of Vh = −60 mV are superimposed. **B**, The NMDA/AMPA ratio in naive (open bar; n = 11) and fear-conditioned (filled bar; n = 20) mice in BLA-CeC synapses. Diamonds indicate individual ratio for each neuron. Sample traces of NMDA current (upper trace) and AMPA current (lower trace) are superimposed for naive (top) and FC (bottom) mice. The average of 15 consecutive waves (5 min) taken 20–25 min after CNQX application, followed by changing holding potential to Vh = +40 mV, and those taken 5 min before the application of CNQX at a holding potential of Vh = −60 mV are superimposed.

To further explore postsynaptic mechanisms underlying the synaptic potentiation of the two pathways, we next examined the effects of FC on the ratio of *N*-methyl-D-aspartate (NMDA) receptor-mediated EPSCs to α-amino-3-hydroxy-5-methyl-4-isoxazolepropionic acid (AMPA) receptor-mediated EPSCs at both synaptic inputs. We found that the averaged value of the NMDA/AMPA ratio tended to increase, but this tendency did not reach statistical significance at either lPB-CeC synapses (29.78 ± 4.23% for naive mice and 35.82 ± 3.58% for FC mice, n = 17 and 21, respectively; *p* = 0.28; Figure 5A) or BLA-CeC synapses (33.69 ± 6.06% for naive mice and 46.94 ± 6.54% for FC mice, n = 11 and 10, respectively; *p* = 0.15; Figure 5B). The lack of an effect of fear conditioning on NMDA/AMPA ratios suggests the potentiation of lPB and BLA synapses onto CeC neurons in slices from fear-conditioned mice may, at least in part, be due to presynaptic changes that affect both components of the EPSCs equally. Alternatively, an enhancement of postsynaptic NMDAR function that preserves NMDA/AMPA ratios at potentiated synapses (see Watt et al., 2004) might also be involved.

**Figure 6:**
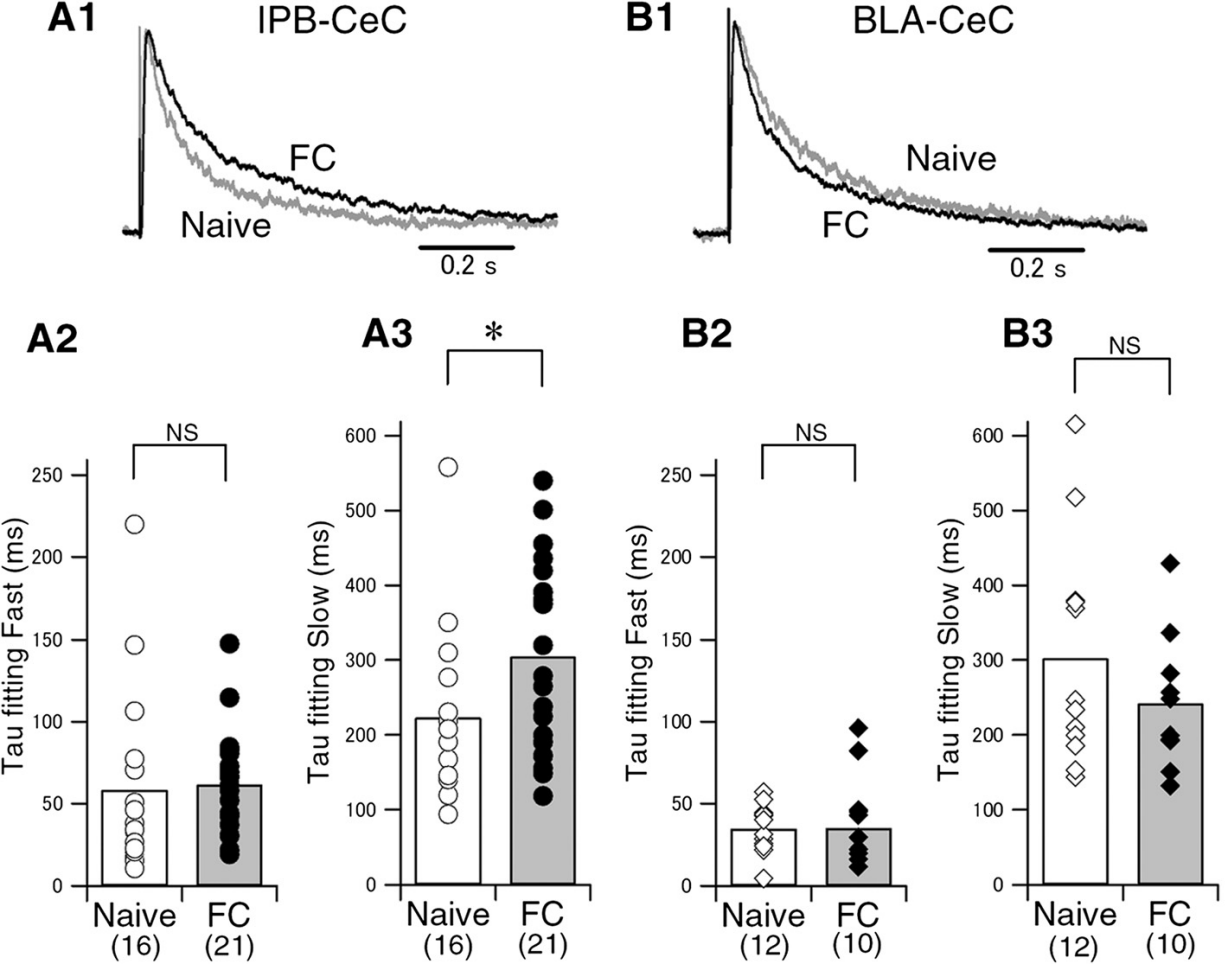
**Kinetics of NMDA receptor-mediated EPSCs in lPB-CeC and BLA-CeC pathways. A1**, Representative traces of scaled NMDA receptor-mediated EPSCs recorded at +40 mV in the presence of CNQX from naive (gray line) and FC (solid line) mice at lPB-CeC synapses. The averaged fast (**A2**) and slow (**A3**) decay time constants of NMDA receptor-mediated EPSCs at lPB-CeC synapses from naive (open bar) and FC (filled bar) mice. Individual values for all the cells are superimposed on the bars. **B1**, Representative traces of scaled NMDA receptor-mediated EPSCs recorded at +40 mV in the presence of CNQX from naive (gray line) and FC (solid line) mice at BLA-CeC synapses. Averaged fast (**B2**) and slow (**B3**) decay time constants of NMDA receptor-mediated EPSCs at BLA-CeC synapses from naive (open bar) and FC (filled bar) mice. Note that at lPB-CeC synapses, the slow decay time constant was significantly increased in FC mice compared with naive mice.

Although NMDA/AMPA ratios were not changed following fear conditioning, we did observe alterations in the kinetics of NMDA-mediated EPSCs in slices from fear-conditioned mice. The decay in the EPSCs measured at a membrane potential of +40 mV in the presence of CNQX was best fitted with double exponential time constants (Figure 6A1, B1) as previously reported [30], with τ_fast_ = 58.4 ± 14.1 msec and τ_slow_ = 223.4 ± 28.2 msec in naive mice at lPB-CeC synapses (n = 16), and τ_fast_ = 61.6 ± 6.6 msec and τ_slow_ = 305.5 ± 28.0 msec in FC mice at lPB-CeC synapses (n = 21; Figure 6A2 and A3). The τ_slow_ was significantly larger (*p* < 0.05) in lPB-CeC synapses in FC mice than in naive mice, suggesting that different synaptic NMDA receptor subunits and/or phosphorylation states might play a role in fear learning. We found no significant differences in the kinetics of the NMDA receptor-mediated EPSC at BLA-CeC synapses between FC and naive mice, with τ_fast_ = 34.6 ± 4.2 msec and τ_slow_ = 302.4 ± 43.3 msec in naive mice (n = 12), and τ_fast_ = 41.1 ± 8.9 msec and τ_slow_ = 242.2 ± 28.4 msec in FC mice (n = 10; Figure 6B2 and B3). The larger decay time constant of the slow decay component may reflect a contribution of GluN2B subunits [30-32]. Further study is needed to clarify the nature of any NMDA receptor component change following fear learning.

### Quantal postsynaptic responses underlying the potentiation of lPB-CeC and BLA-CeC synapses

**Figure 7:**
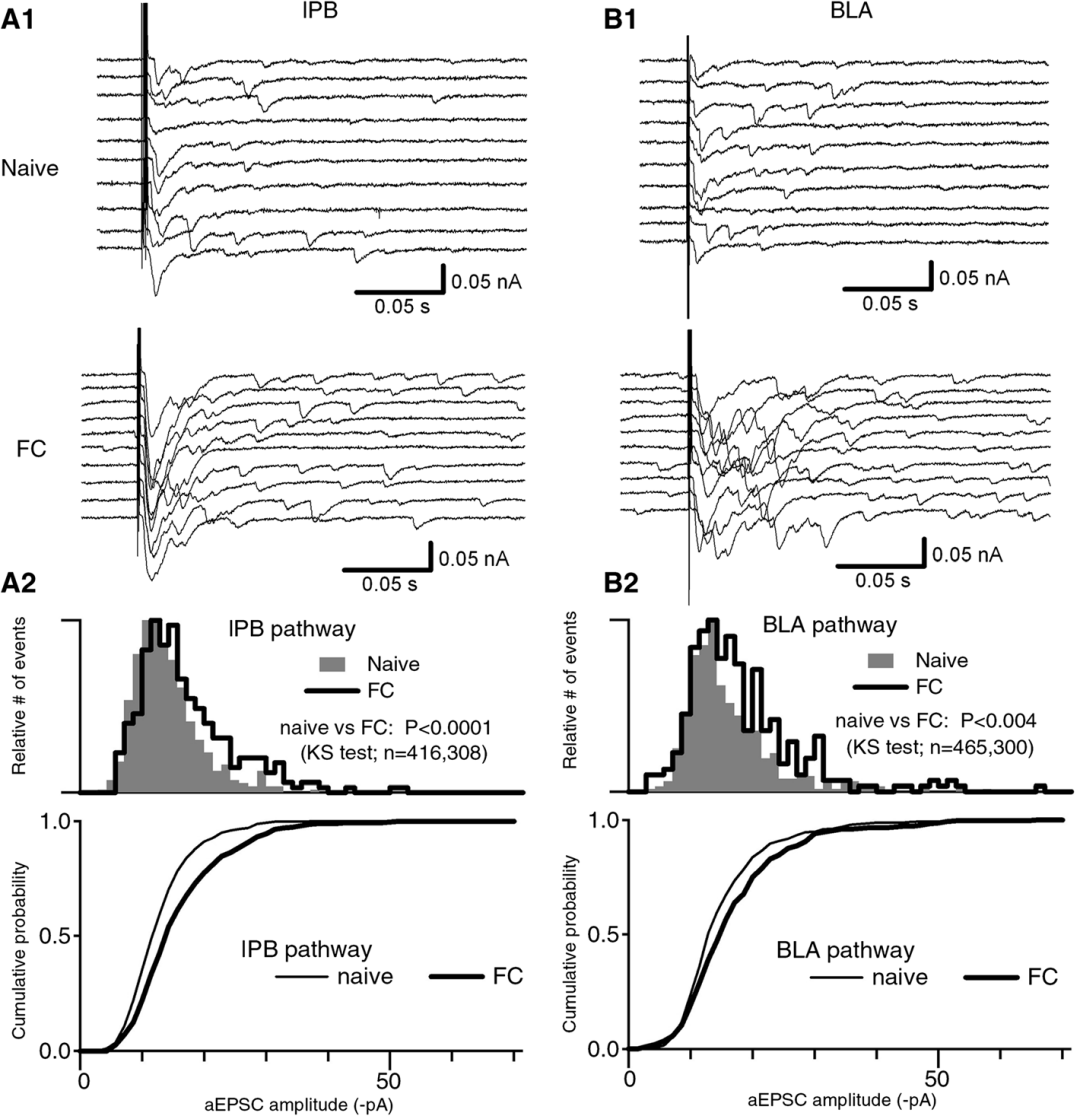
**Asynchronous EPSC amplitudes in lPB-CeC and BLA-CeC synapses. A1 ***and ***B1**, Representative traces using strontium solution (5 mM Sr^2+^, 0 mM Ca^2+^) in lPB-CeC (A1) and BLA-CeC (B1) synapses from naive (top panels) and FC (bottom panels) mice. **A2***and***B2**, Histograms (top panels) and cumulative plots (bottom panels) of the asynchronous EPSC (aEPSC) amplitudes in lPB-CeC (A2) and BLA-CeC (B2) synapses. Note that aEPSCs with larger amplitude emerged in FC mice so that the relative amplitude histograms were skewed towards the right. The distribution was significantly different in both lPB-CeC (*p* < 0.0001) and BLA-CeC (*p* < 0.004) synapses.

To better define the potential presynaptic and postsynaptic contributions to fear conditioning-induced potentiation at synapses onto CeC neurons, we investigated the quantal amplitude of synaptic inputs by analyzing asynchronous events evoked in the presence of 5 mM strontium, which replaced the extracellular calcium [33]. These asynchronous EPSCs (aEPSCs) were separately analyzed for the lPB and BLA pathways. Figure 7 A1 and B1 show representative traces, in which events with larger amplitude were recorded from FC mice compared with naive mice. The distribution of the aEPSC amplitude histogram was skewed towards larger amplitude in neurons from FC mice, compared with naive mice, at lPB-CeC synapses (308 events in 5 cells from 2 FC mice, and 416 events in 6 cells from 3 naive mice, *p* < 0.0001, KS test; Figure 7A2) as well as at BLA-CeC synapses (300 events in 5 cells from 2 FC mice, and 464 events in 6 cells from 3 naive mice, *p* < 0.004, KS test; Figure 7B2). These results indicate that quantal responses with larger amplitude emerged in both lPB-CeC and BLA-CeC synapses after fear learning, suggesting an important role of enhanced postsynaptic AMPAR function in the potentiation of these two pathways.

### Nociceptive thresholds in fear-conditioned mice

**Figure 8:**
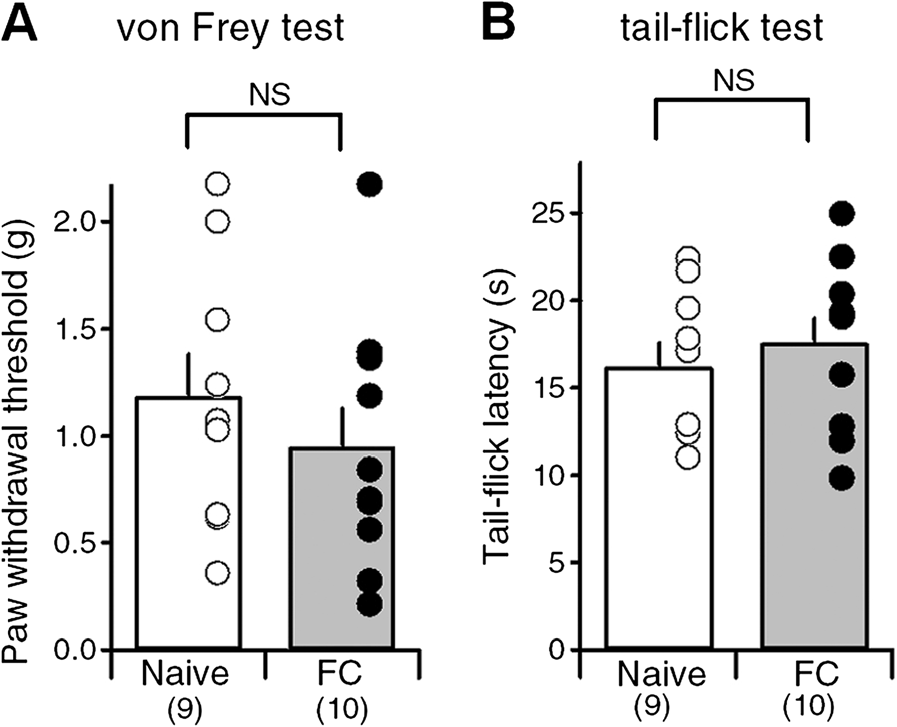
**Mechanical and thermal nociceptive threshold in naive and FC mice. A**, Averaged paw withdrawal threshold measured by von Frey filament test in naive (open bar) and FC (filled bar) mice. Individual values from all the mice are superimposed on each bar. **B**, Averaged response latencies in the tail-flick test in naive (open bar) and FC (filled bar) mice. Note that neither mechanical nor thermal nociceptive threshold differed between naive and FC mice.

The lPB-CeC synapses are also known to express nociception-induced plasticity [14,25,34,35], which can modulate nocifensive behaviors by modifying nociceptive threshold, probably via descending regulatory pathways [24]. In this study, we employed a rather stronger protocol for fear conditioning to increase the likelihood of activating more CeC neurons. This might have developed a nociception-induced plasticity in nociceptive threshold. To examine this possibility, we evaluated changes in the nociceptive threshold by estimating the paw withdrawal threshold and the thermal tail-flick response. We found no significant changes in the nociceptive threshold against mechanical (Figure 8A) or thermal (Figure 8B) stimulation in the fear-conditioned mice.

## Discussion

It is widely accepted that LA pyramidal neurons play a significant role in the association of CS and US during fear memory formation [2,36-38]. However, accumulating data suggest that other brain regions including the CeA [3,4,39], also have an important role in the acquisition of fear memory. Our results showing that lPB and BLA synapses onto CeC neurons are potentiated following fear learning indicate that the CeC likely represents another important site for the association of CS and US signals in fear learning. Thus, during the sequential flow of information from the LA to the BLA to the CeC/CeL to the CeM, polymodal CS information may be integrated and associated with US-related information at each step in this pathway. This suggests that, in addition to the LA, there may be multiple sites in this serial processing circuit, such as in the CeC, where CS-US associations can occur and that such a multi-site integration system may allow robust and modality-specific regulation of fear learning.

### Molecular mechanisms underlying synaptic plasticity

The decreased PPR at BLA-CeC and lPB-CeC synapses in cells from fear-conditioned mice indicates that increases in presynaptic vesicle release probability contribute to the potentiation of these synapses following fear conditioning. We also found, however, that the distribution of aEPSC amplitude was significantly skewed towards larger amplitudes in FC mice compared with naive animals (Figure 7). This indicates that the number and/or activity of postsynaptic AMPA receptor is also enhanced following fear learning. Thus, both presynaptic and postsynaptic changes appear to contribute substantially to the strengthening of these synapses in fear conditioning. Consistent with this notion, the kinetics of NMDA receptor-mediated EPSCs were significantly altered in the lPB-CeC pathway (Figure 6A3), suggesting that a change in subunit composition and/or other modifications may have occurred at least in the lPB-CeC synapses. Taken together, the present findings demonstrate that the synaptic potentiation is mediated by an increase in vesicular release probability together with postsynaptic AMPA receptor modulation, which might also accompany the potentiation of NMDA receptors. These results are of particular interest because BLA and lPB inputs form synapses onto different dendritic sites in CeC neuron; the BLA inputs form synapses onto the dendritic spines, while the lPB inputs do so on the dendritic shafts [27]. The correlation between BLA-CeC and lPB-CeC synaptic potentiation suggests some heterosynaptic interaction between these two distinct pathways. The most plausible mechanisms might include postsynaptic spine-to-shaft or shaft-to-spine interactions. For example, synaptic potentiation in the BLA-CeC pathway might be integrated and associated further at the CeC through the lPB-CeC pathway, resulting in heterosynaptic potentiation of the lPB-CeC synapses. Alternatively, lPB-CeC synaptic potentiation by nociceptive associative learning would intensify its instructive function in heterosynaptic plasticity, possibly leading to an additional potentiation of BLA-CeC synapses. The non-significant BLA-CeC potentiation following fear conditioning without retrieval (i.e., FC alone group) may suggest such lPB to BLA interaction in plasticity regulation. One of the plausible interpretations is that a slective activation of BLA-CeC synaptic transmission by thalamocortical inputs, such as that seen in retrievals, would be one of the factors that help maintaining the potentiation.

### BLA-CeC synaptic potentiation following fear learning

In the present study, we identified BLA-CeC potentiation following fear learning. In contrast, a previous study reported that BLA-CeL synapses were not potentiated after fear learning in rats [6]. Differences in experimental protocols might account for this discrepancy. For example, Amano et al. examined synaptic transmission 48 h after conditioning, while we examined synaptic transmission 24 h after conditioning. Thus, one possibility is that the potentiation of BLA synaptic inputs onto neurons in the central nucleus is transient and decays with a slow time course post-conditioning. Also, animals were conditioned using 4-pairings in the experiments of Amano et al. while 9-pairings were employed in our experiments. Thus, another possibility is that extended training may enhance the potentiation or the ability to detect potentiation of BLA inputs simply by recruiting a larger population of CeC neurons during conditioning. Finally, Amano et al. recorded from the CeL, which may include both the CeL and CeC judged by our criterion, while our recordings focused mainly on neurons in the CeC region. Thus, fear conditioning may have different effects on BLA synaptic inputs onto neurons in distinct subdivisions of the central nucleus. In support of this notion, previous detailed analysis revealed that CeC and CeL neurons target different areas within the amygdala [5]. Furthermore, projection of lPB-arising fibers to CeA shows a latero-medial gradient [27], which is accompanied with distinct morphological properties in the presynaptic terminals [16]. It is interesting, however, that Amano et al. (2010) reported that BLA-CeL synapses are potentiated by unpaired presentations of the US and CS, suggesting that plasticity at these synapses may be involved in contextual fear conditioning. It would be intriguing to pursue dissociation between BLA-CeC and lPB-CeC synaptic potentiation in a future study.

While the increased release probability after fear conditioning in this study is reminiscent of NMDA receptor-independent presynaptic LTP following tetanic stimulation at lPB-CeL synapses [26], the lPB-CeC potentiation observed in this study may not be fully regulated by the same mechanism as lPB-CeL synapses.

### CeC/CeL and CeM inhibitory network regulation in fear learning

Previous studies reported that CeM cells are tonically inhibited by CeL/CeC cells at the basal state, and disinhibition of these synapses is involved in fear learning [9,10]. Ciocchi et al. demonstrated that the CeL/CeC contains at least two functionally distinct neuronal subpopulations, each exhibiting opposite response to CS presentation: CS_off_ neurons and CS_on_ neurons, which regulate CS_off_ neurons. An intriguing speculation is that the majority of CeC neurons examined in the present study may correspond to CS_on_ neurons, provided a latero-medial gradient in the distribution of CS_on_ and CS_off_ neurons over the CeC and CeL regions. Although the use of cesium-containing internal solution in the present study prevented the identification of firing patterns, which might have allowed us to distinguish type I vs. type II, or CS_off_ vs. CS_on_ neurons [9,10,18], a plausible interpretation of the present results is that CeC neurons are involved in disinhibition by orchestrating tonic as well as phasic inhibition of CeM neurons through the regulation of inhibitory microcircuits under the synaptic control of BLA and lPB pathway inputs.

### Physiological consequences of synaptic potentiation at the CeC

A large number of literatures suggest relationship between the fear learning and the pain regulation. It is well established that the fear memory recall with cues and/or contexts in the fear-conditioned animals attenuate nociceptive behaviors, which is termed as conditioned fear analgesia. It is postulated that shared CNS regions including the amygdala and periaqueductal grey underlie such interaction [40-42]. In contrast, in the present study, the nociceptive threshold was measured in the absence of fear cues to measure the nociceptive threshold at the spinal level in isolation. Therefore, our data simply demonstrate that, at least at 24 h after FC, the enhanced connectivity of spino-parabrachio-amygdaloid pathway is not accompanied by enhanced spinal nociceptive reflex (Figure 8A, 8B). It is therefore an interesting future subject whether such synaptic enhancement after fear learning would result in development of enhanced pain-induced behavior and/or lowered pain threshold in later phases. The results of such study would provide etiological basis for the significantly higher incidents of PTSD in the chronic pain patients [43].

Pain is an emotional state whereby noxious stimuli detected and processed by nociceptors are integrated and associated with negative affective factors. The CeC plays a pivotal role in associating this emotional component with nociceptive signals, by integrating direct nociceptive signals from the lPB with highly processed polymodal signals from the BLA. Thus, one possibility is that lPB-CeC synaptic potentiation is not the underlying mechanism of fear learning *per se*, but rather a consequence of fear learning; that is, this potentiation provides a trace of noxious experience, thereby allowing animals to more readily associate BLA signals with concurrent lPB signals that convey nociceptive/aversive information. Such metaplastic mechanisms might regulate the threshold and/or efficacy of subsequent fear learning [44]. Another possible physiological consequence of the synaptic potentiation of the lPB-CeC pathway might be fear generalization, in which CS specificity is compromised so that conditioned fear responses are generalized to tones other than the one paired with US [45,46]. It may be useful for future studies to examine the causality between synaptic potentiation and its behavioral consequences using molecular and/or optogenetic approaches to clarify these issues in detail.

The multi-step mode of synaptic plasticity observed in the present study functions in an organized manner, allowing highly processed multimodal signals in the LA to be integrated and further associated with negative affective information in the CeC. A report appearing after the submission of this paper indicated a synaptic potentiation of LA input onto somatostatin-positive cells in CeL following fear conditioning in mice [47]. This highly convincing work is also an example supportive of the notion that fear-related associative memory is borne at multiple steps in the amygdaloid information traffic among LA, BLA, CeC, CeL and CeM. As each of these structures receives distinct neuronal inputs from diverse origins and also is under strong influence of distinct chemical modulators such as monoamines and neuropeptides, it is conceivable that such multi-step regulation system would allow more robust optimization of the behavioral program in response to aversive information.

## Conclusions

In summary, the present study demonstrates synaptic potentiation of both lPB-CeC and BLA-CeC pathways following fear learning in an associative manner, which is mediated by both presynaptic and postsynaptic mechanisms. These results suggest that the CeC may provide another locus of CS-US association, in addition to the LA, in fear memory formation.

## Methods

The manipulation of the animals was approved by the Institutional Committee for the Care and Use of Experimental Animals of The Jikei University School of Medicine (Approval No. 21-061C5). All experiments were conformed to the Guidelines for Proper Conduct of Animal Experiments of the Science Council of Japan (2006) and to the guidelines of the International Association for the Study of Pain (Zimmermann 1983).

### Fear conditioning

Male C57BL/6 J mice (5–6 weeks old) (CLEA Japan Inc., Tokyo, Japan) were group-housed under a 12-h light/dark cycle, and provided with food and water *ad libitum*. Animals were habituated to handling for more than 7 days before being divided into five groups (Figure 1A); naive, fear-conditioned (FC), FC alone, CS alone and unpaired groups. All the conditioning procedures were conducted in a conditioning chamber (170 mm width × 100 mm depth × 100 mm height) surrounded by a sound-attenuating chamber (CL-M3, O’Hara & Co., Ltd., Tokyo, Japan). For the FC group, mice received nine presentations of tone CS (10 kHz, 65 dB, 20 s), each terminating with a foot shock US (0.6 mA, 2 s), presented in a conditioning chamber (200 Lux, 50 dB background white noise). The first CS was delivered 120 s after the animal was placed in the chamber, and inter-trial intervals were pseudo-randomly selected, ranging from 40 to 480 s. A retrieval trial was performed 24 h later in a novel chamber (with a white acrylic plate wall washed with peppermint-scented soap). The CS presentation began 120 s after the mice were placed in the chamber, and lasted for 120 s (50 Lux, 60 dB background white noise). Mice in the FC alone group received exactly the same fear conditioning as FC mice, but were not subjected to the retrieval tests. Mice in the CS alone group were treated similarly to the FC group, with the exception that they received only tone CS, with no accompanying US, during the conditioning. For the unpaired group, the mice received nine US immediately after being placed into the conditioning chamber with 2-s intervals, followed by nine CS presentations with 5-s intervals. Freezing behavior was measured using a digital camera connected to a computer running Time FZ1 (O’Hara & Co., Ltd), a software package based on NIH Image, which was calibrated for the counting of freezing behavior by C57BL/6 mice using scoring performed by two independent human observers prior to the actual experiments. Slice preparation was performed approximately 15 min after the end of retrieval for the FC, CS alone and unpaired groups, and 24 h after conditioning for the FC alone group. Mice in the naive group were handling-habituated only followed by slice preparation.

### Amygdala slice preparation and patch-clamp recording

The mice were anesthetized with isoflurane (5%) and sacrificed. The brain was removed and blocks containing the amygdala were prepared in ice-cold cutting solution containing (in mM) NaCl 125, KCl 3, CaCl_2_ 0.1, MgCl_2_ 5, NaH_2_PO_4_ 1.25, D-glucose 10, L-ascorbic acid 0.4 and NaHCO_3_ 25 (pH 7.4) equilibrated with 95% O_2_/5% CO_2_. Transverse brain slices, 400 μm thick, containing the central amygdala were prepared using a vibrating blade slicer (VT1200S, Leica) at 0°C, transferred to a chamber containing ACSF ([in mM] NaCl 125, KCl 3, CaCl_2_ 2, MgCl_2_ 1.3, NaH_2_PO_4_ 1.25, D-glucose 10, L-ascorbic acid 0.4 and NaHCO_3_ 25 [pH 7.4]) and incubated in an atmosphere of 95% O_2_/5% CO_2_ at 37°C for 30 min, then maintained for several hours in ACSF at room temperature. Neurons in the latero-capsular division of the CeA were visually identified using oblique illumination optics microscopy (BX51WI, Olympus) and a charge-coupled device camera (IR-1000, DageMTI),. Each coronal slice was matched with the corresponding rostrocaudal level of Paxinos and Franklin [8]. Whole-cell recordings were made from brain slices maintained at 30 ± 2°C in a recording chamber continuously perfused with oxygenated ACSF (95% O_2_/5% CO_2_) at a flow rate of 1.5-2.0 ml/min. The tip resistance of the recording electrodes was 4–9 MΩ, and the recording electrodes were filled with internal solution containing (in mM) Cs-gluconate 122.5, CsCl 17.5, NaCl 8, HEPES 10, EGTA 0.2, ATP magnesium 2, GTP sodium 0.3 (pH 7.2; osmolarity, 290–310 mOsm).

With custom-designed bipolar parallel stimulation electrodes (TOG211-039a, Unique Medical Co., Ltd., Tokyo, Japan), EPSCs were evoked in CeC neurons by electrical stimulation of the two afferent synapses to the CeC: the lPB and BLA pathways under microscopic control (Figure 1C, D) as described previously [25,29]. The lPB-stimulating electrode was placed onto the fibers that run dorsomedial to the CeA and ventral to, but outside of, the caudate-putamen. BLA-stimulating electrode was placed in the ventral BLA near the borderline to the CeA [25,29,46]. Picrotoxin (100 μM) was present in the ACSF to isolate EPSCs. EPSCs were recorded at a holding potential of −60 mV with a patch-clamp amplifier (MultiClamp 700B, Molecular Devices, Foster City, CA), low-pass filtered at 2 kHz and sampled at 10 kHz at a 16-bit resolution with a PowerLab interface (AD Instruments) and pClamp 10 software (Molecular Devices). The series resistance was constantly monitored, and data were discarded if they varied more than 20% within an experiment.

To calculate the paired-pulse ratio (PPR) of EPSCs, two pulses with an interstimulus interval of 100 ms were delivered, except for the cross-pathway PPR experiments in which 50-ms interval was applied. The PPR was calculated as normalized amplitude EPSC_2nd_/EPSC_1st_.

When NMDA receptor-mediated EPSCs were recorded, 10 μM 6-cyano-7-nitroquinoxaline-2,3-dione (CNQX) was bath-applied to block AMPA-type glutamate receptor-mediated EPSCs and the holding potential was kept at +40 mV. To analyze NMDA receptor-mediated EPSC kinetics, we averaged 15 consecutive EPSCs, and the current decay was fitted using a previously reported double-exponential equation as follows:

$$I_{t}\;=\;I_{f}\;\exp\left({-t/\tau }_{\mathrm{fast}}\right)\;+\;I_{t}\;\exp\left({-t/\tau }_{\mathrm{slow}}\right)$$

To isolate asynchronous EPSCs (aEPSCs), extracellular Ca^2+^ (2 mM) was replaced with Sr^2+^ (5 mM) after confirming stable postsynaptic responses evoked by the stimulation of lPB-CeC and BLA-CeC pathways. The amplitude of aEPSC was evaluated between 20 ms and 120 ms poststimulus to exclude synchronously released evoked events. The recordings were obtained for five minutes after confirming the external Ca^2+^–containing solution was fully replaced by Sr^2+^-containing solution for more than 15 min.

Cross-pathway PPR experiments were conducted in naive male C57BL/6 J mice (7–8 weeks old) to examine the possible overlap between the fibers stimulated by the lPB-stimulating and BLA-stimulating electrodes. Baseline EPSCs were obtained using a paired-pulse protocol in each pathway (single-pathway PPR; 50-ms inter-pulse interval, 0.05 Hz, stimulation of the two pathways were separated by 10 s). Following stable baseline, cross-pathway PPR between lPB-CeC pathway and BLA-CeC pathway was measured by stimulating lPB-CeC pathway 50 ms after BLA-CeC pathway, or *vice versa*. Cross-pathway PPR is defined as EPSC _2nd BLA of cross-path PPR (lPB-BLA)_/EPSC _1st BLA of single-path PPR (BLA-BLA)_ for lPB effect on BLA pathway, and EPSC _2nd lPB of cross-path PPR (BLA-lPB)_/EPSC _1st lPB of single-path PPR (lPB-lPB)_ for BLA effect on lPB pathway.

### Evaluation of nociceptive responses

The paw withdrawal threshold to mechanical stimuli was evaluated by experienced experimenters, according to a previously reported method [48]. Mechanical stimuli were applied using von Frey filaments of different rigidity (0.02-2.0 g). Each mouse was placed on a metal mesh floor, and a von Frey filament was applied manually from beneath. The 50% threshold was estimated using the up-and-down method [49]. The mice were allowed to habituate in the 500-ml glass beaker placed up-side down on the mesh floor at least for 30 min prior to the experiments. Judgment of the paw withdrawal reflex was done by experienced examinators to avoid unnecessary re-examination. The results were compared “on-site” with the estimation table for 50%-threshold and the test was terminated once the minimum necessary data for estimation was attained. The mechanical threshold was determined as the average of both hindpaw measurements per mouse.

The thermal nociceptive response was evaluated by recording the latency to withdrawal of the tail in response to noxious skin heating. Briefly, the tails of mice were exposed to a focused beam of light from a 50-W projection bulb. The beam intensity was set to produce a temperature of 75°C using a tail-flick analgesia meter MK-330B (Muromachi Kikai Co., Ltd., Tokyo, Japan). In the absence of a response within a predetermined maximum latency (30 s), the trial was terminated to prevent tissue damage*.*

### Data and statistical analysis

The recorded membrane current was analyzed off-line using an Igor Pro 5 (WaveMetrics, OR, USA) using macros written by one of the authors (F.K.). Peak amplitude was measured on the basis of the averaged waveform of evoked EPSCs (five consecutive trials). Values are expressed as mean ± standard error of the mean (SEM). Statistical analysis consisted of analysis of variance (ANOVA) followed by *post hoc* Dunnett’s test, Student’s *t*-test, Kolmogorov-Smirnov (KS) test or Mann–Whitney *U* test. Differences with a *p*-value less than 0.05 were considered significant.

## Abbreviations

CeA: Central nucleus of the amygdala; CeC: The capsular division of CeA; lPB: External part of the lateral parabrachial nucleus; BLA: Basolateral nucleus of the amygdala; EPSC: Excitatory postsynaptic current; NMDA: *N*-methyl-D-aspartate; AMPA: α-amino-3-hydroxy-5-methyl-4-isoxazolepropionic acid.

## Competing interests

The authors declare no conflict of interest.

## Authors’ contributions

The research was designed by AMW and FK and performed in the laboratory of FK. The experiments were performed by AMW, TO, and MN. The data were analyzed by AMW, TO, MN, and FK with assistance of YT and MS. The manuscript was written by AMW and FK All authors have read and approved the final version of the manuscript.

## References

[B1] Davis M Whalen PJ The amygdala: vigilance and emotion Mol Psychiatry 2001 6 1 13 34 10.1038/sj.mp.4000812 11244481

[B2] LeDoux JE Emotion circuits in the brain Annu Rev Neurosci 2000 23 155 184 10.1146/annurev.neuro.23.1.155 10845062

[B3] Wilensky AE Schafe GE Kristensen MP LeDoux JE Rethinking the fear circuit: the central nucleus of the amygdala is required for the acquisition, consolidation, and expression of Pavlovian fear conditioning J Neurosci 2006 26 48 12387 12396 10.1523/JNEUROSCI.4316-06.2006 17135400 PMC6674909

[B4] Zimmerman JM Rabinak CA McLachlan IG Maren S The central nucleus of the amygdala is essential for acquiring and expressing conditional fear after overtraining Learn Mem 2007 14 9 634 644 10.1101/lm.607207 17848503 PMC1994080

[B5] Busti D Geracitano R Whittle N Dalezios Y Manko M Kaufmann W Satzler K Singewald N Capogna M Ferraguti F Different fear states engage distinct networks within the intercalated cell clusters of the amygdala J Neurosci 2011 31 13 5131 5144 10.1523/JNEUROSCI.6100-10.2011 21451049 PMC6622967

[B6] Amano T Unal CT Pare D Synaptic correlates of fear extinction in the amygdala Nat Neurosci 2010 13 4 489 494 10.1038/nn.2499 20208529 PMC2847017

[B7] Ehrlich I Humeau Y Grenier F Ciocchi S Herry C Luthi A Amygdala inhibitory circuits and the control of fear memory Neuron 2009 62 6 757 771 10.1016/j.neuron.2009.05.026 19555645

[B8] Paxinos GF Franklin KBJ The mouse brain in stereotaxic coordinates 2004 compact second San Diego: Academic Press

[B9] Ciocchi S Herry C Grenier F Wolff SB Letzkus JJ Vlachos I Ehrlich I Sprengel R Deisseroth K Stadler MB Encoding of conditioned fear in central amygdala inhibitory circuits Nature 2010 468 7321 277 282 10.1038/nature09559 21068837

[B10] Haubensak W Kunwar PS Cai H Ciocchi S Wall NR Ponnusamy R Biag J Dong HW Deisseroth K Callaway EM Genetic dissection of an amygdala microcircuit that gates conditioned fear Nature 2010 468 7321 270 276 10.1038/nature09553 21068836 PMC3597095

[B11] Duvarci S Popa D Pare D Central amygdala activity during fear conditioning J Neurosci 2011 31 1 289 294 10.1523/JNEUROSCI.4985-10.2011 21209214 PMC3080118

[B12] Bernard JF Alden M Besson JM The organization of the efferent projections from the pontine parabrachial area to the amygdaloid complex: a Phaseolus vulgaris leucoagglutinin (PHA-L) study in the rat J Comp Neurol 1993 329 2 201 229 10.1002/cne.903290205 8454730

[B13] Bernard JF Bandler R Parallel circuits for emotional coping behaviour: new pieces in the puzzle J Comp Neurol 1998 401 4 429 436 10.1002/(SICI)1096-9861(19981130)401:4<429::AID-CNE1>3.0.CO;2-3 9826271

[B14] Neugebauer V Li W Differential sensitization of amygdala neurons to afferent inputs in a model of arthritic pain J Neurophysiol 2003 89 2 716 727 12574449 10.1152/jn.00799.2002

[B15] Todd AJ Neuronal circuitry for pain processing in the dorsal horn Nat Rev Neurosci 2010 11 12 823 836 21068766 10.1038/nrn2947 PMC3277941

[B16] Sarhan M Freund-Mercier M-J Veinante P Branching patterns of parabrachial neurons projecting to the central extended amgydala: Single axonal reconstructions J Comp Neurol 2005 491 4 418 442 10.1002/cne.20697 16175547

[B17] Fanselow MS Gale GD The amygdala, fear, and memory Ann N Y Acad Sci 2003 985 125 134 12724154 10.1111/j.1749-6632.2003.tb07077.x

[B18] Sah P Faber ES Lopez De Armentia M Power J The amygdaloid complex: anatomy and physiology Physiol Rev 2003 83 3 803 834 12843409 10.1152/physrev.00002.2003

[B19] Pare D New vistas on amygdala networks in conditioned fear J Neurophysiol 2004 92 1 1 9 10.1152/jn.00153.2004 15212433

[B20] Phelps EA LeDoux JE Contributions of the amygdala to emotion processing: from animal models to human behavior Neuron 2005 48 2 175 187 10.1016/j.neuron.2005.09.025 16242399

[B21] Shi C Davis M Pain pathways involved in fear conditioning measured with fear-potentiated startle: lesion studies J Neurosci 1999 19 1 420 430 9870970 10.1523/JNEUROSCI.19-01-00420.1999 PMC6782355

[B22] Cheng SJ Chen CC Yang HW Chang YT Bai SW Yen CT Min MY Role of extracellular signal-regulated kinase in synaptic transmission and plasticity of a nociceptive input on capsular central amygdaloid neurons in normal and acid-induced muscle pain mice J Neurosci 2011 31 6 2258 2270 10.1523/JNEUROSCI.5564-10.2011 21307262 PMC6633051

[B23] Neugebauer V Li W Processing of nociceptive mechanical and thermal information in central amygdala neurons with knee-joint input J Neurophysiol 2002 87 1 103 112 11784733 10.1152/jn.00264.2001

[B24] Carrasquillo Y Gereau RW Activation of the extracellular signal-regulated kinase in the amygdala modulates pain perception J Neurosci 2007 27 7 1543 1551 10.1523/JNEUROSCI.3536-06.2007 17301163 PMC6673749

[B25] Ikeda R Takahashi Y Inoue K Kato F NMDA receptor-independent synaptic plasticity in the central amygdala in the rat model of neuropathic pain Pain 2007 127 1–2 161 172 17055162 10.1016/j.pain.2006.09.003

[B26] Lopez de Armentia M Sah P Bidirectional synaptic plasticity at nociceptive afferents in the rat central amygdala J Physiol 2007 581 Pt 3 961 970 17379642 10.1113/jphysiol.2006.121822 PMC2170827

[B27] Dong Y-L Fukazawa Y Wang W Kamasawa N Shigemoto R Differential postsynaptic compartments in the laterocapsular division of the central nucleus of amygdala for afferents from the parabrachial nucleus and the basolateral nucleus in the rat J Comp Neurol 2010 518 23 4771 4791 10.1002/cne.22487 20963828

[B28] Xu-Friedman MA Regehr WG Structural contributions to short-term synaptic plasticity Physiol Rev 2004 84 1 69 85 10.1152/physrev.00016.2003 14715911

[B29] Neugebauer V Li W Bird GC Bhave G Gereau RW Synaptic plasticity in the amygdala in a model of arthritic pain: differential roles of metabotropic glutamate receptors 1 and 5 J Neurosci 2003 23 1 52 63 12514201 10.1523/JNEUROSCI.23-01-00052.2003 PMC6742141

[B30] Lopez de Armentia M Sah P Development and subunit composition of synaptic NMDA receptors in the amygdala: NR2B synapses in the adult central amygdala J Neurosci 2003 23 17 6876 6883 12890782 10.1523/JNEUROSCI.23-17-06876.2003 PMC6740716

[B31] Vicini S Wang JF Li JH Zhu WJ Wang YH Luo JH Wolfe BB Grayson DR Functional and pharmacological differences between recombinant N-methyl-D-aspartate receptors J Neurophysiol 1998 79 2 555 566 9463421 10.1152/jn.1998.79.2.555

[B32] Tovar KR Westbrook GL The incorporation of NMDA receptors with a distinct subunit composition at nascent hippocampal synapses *in vitro* J Neurosci 1999 19 10 4180 4188 10234045 10.1523/JNEUROSCI.19-10-04180.1999 PMC6782704

[B33] Goda Y Stevens CF Two components of transmitter release at a central synapse Proc Natl Acad Sci USA 1994 91 26 12942 12946 10.1073/pnas.91.26.12942 7809151 PMC45556

[B34] Han JS Neugebauer V Synaptic plasticity in the amygdala in a visceral pain model in rats Neurosci Lett 2004 361 1–3 254 257 15135941 10.1016/j.neulet.2003.12.027

[B35] Bird GC Lash LL Han JS Zou X Willis WD Neugebauer V Protein kinase A-dependent enhanced NMDA receptor function in pain-related synaptic plasticity in rat amygdala neurones J Physiol 2005 564 Pt 3 907 921 15760935 10.1113/jphysiol.2005.084780 PMC1464474

[B36] Tsvetkov E Carlezon WA Benes FM Kandel ER Bolshakov VY Fear conditioning occludes LTP-induced presynaptic enhancement of synaptic transmission in the cortical pathway to the lateral amygdala Neuron 2002 34 2 289 300 10.1016/S0896-6273(02)00645-1 11970870

[B37] Maren S Quirk GJ Neuronal signalling of fear memory Nat Rev Neurosci 2004 5 11 844 852 10.1038/nrn1535 15496862

[B38] Rumpel S LeDoux J Zador A Malinow R Postsynaptic receptor trafficking underlying a form of associative learning Science 2005 308 5718 83 88 10.1126/science.1103944 15746389

[B39] Johansen JP Tarpley JW LeDoux JE Blair HT Neural substrates for expectation-modulated fear learning in the amygdala and periaqueductal gray Nat Neurosci 2010 13 8 979 986 10.1038/nn.2594 20601946 PMC2910797

[B40] Fanselow MS Helmstetter FJ Conditional analgesia, defensive freezing, and benzodiazepines Behav Neurosci 1988 102 2 233 243 3365319 10.1037//0735-7044.102.2.233

[B41] Helmstetter FJ Bellgowan PS Lesions of the amygdala block conditional hypoalgesia on the tail flick test Brain Res 1993 612 1–2 253 257 8330203 10.1016/0006-8993(93)91669-j

[B42] Rea K Roche M Finn DP Modulation of conditioned fear, fear-conditioned analgesia, and brain regional C-Fos expression following administration of muscimol into the Rat basolateral amygdala J Pain 2011 12 6 712 721 10.1016/j.jpain.2010.12.010 21459678

[B43] McWilliams LA Cox BJ Enns MW Mood and anxiety disorders associated with chronic pain: an examination in a nationally representative sample Pain 2003 106 1–2 127 133 14581119 10.1016/s0304-3959(03)00301-4

[B44] Parsons RG Davis M A metaplasticity-like mechanism supports the selection of fear memories: role of protein kinase a in the amygdala J Neurosci 2012 32 23 7843 7851 10.1523/JNEUROSCI.0939-12.2012 22674260 PMC3375025

[B45] Armony JL Servan-Schreiber D Romanski LM Cohen JD LeDoux JE Stimulus generalization of fear responses: effects of auditory cortex lesions in a computational model and in rats Cereb Cortex 1997 7 2 157 165 10.1093/cercor/7.2.157 9087823

[B46] Shaban H Humeau Y Herry C Cassasus G Shigemoto R Ciocchi S Barbieri S van der Putten H Kaupmann K Bettler B Generalization of amygdala LTP and conditioned fear in the absence of presynaptic inhibition Nat Neurosci 2006 9 8 1028 1035 10.1038/nn1732 16819521

[B47] Li H Penzo MA Taniguchi H Kopec CD Huang ZJ Li B Experience-dependent modification of a central amygdala fear circuit Nat Neurosci 2013 16 3 332 339 10.1038/nn.3322 23354330 PMC3581751

[B48] Tsuda M Shigemoto-Mogami Y Koizumi S Mizokoshi A Kohsaka S Salter MW Inoue K P2X4 receptors induced in spinal microglia gate tactile allodynia after nerve injury Nature 2003 424 6950 778 783 10.1038/nature01786 12917686

[B49] Chaplan SR Bach FW Pogrel JW Chung JM Yaksh TL Quantitative assessment of tactile allodynia in the rat paw J Neurosci Methods 1994 53 1 55 63 10.1016/0165-0270(94)90144-9 7990513

